# Repetitive deep TMS in alcohol dependent patients halts progression of white matter changes in early abstinence

**DOI:** 10.1111/pcn.13624

**Published:** 2023-12-12

**Authors:** Mohamed Kotb Selim, Maayan Harel, Silvia De Santis, Irene Perini, Wolfgang H. Sommer, Markus Heilig, Abraham Zangen, Santiago Canals

**Affiliations:** ^1^ Instituto de Neurociencias Consejo Superior de Investigaciones Científicas (CSIC) and Universidad Miguel Hernández (UMH) Sant Joan d'Alacant Spain; ^2^ Department of Life Sciences Ben‐Gurion University Beer Sheva Israel; ^3^ Zlotowski Center for Neuroscience Ben‐Gurion University Beer Sheva Israel; ^4^ Center for Social and Affective Neuroscience, Department of Biomedical and Clinical Sciences Linköping University Hospital Linköping Sweden; ^5^ Department of Addiction Medicine, Department of Clinical Psychology Medical Faculty Mannheim, Central Institute of Mental Health, University of Heidelberg Mannheim Germany

**Keywords:** Addiction Remission Network, Alcohol Use Disorder, Deep TMS, DTI, fMRI

## Abstract

**Aim:**

Alcohol use disorder (AUD) is the most prevalent form of addiction, with a great burden on society and limited treatment options. A recent clinical trial reported significant clinical benefits of deep transcranial magnetic stimulations (Deep TMS) targeting midline frontocortical areas. However, the underlying biological substrate remained elusive. Here, we report the effect of Deep TMS on the microstructure of white matter.

**Methods:**

A total of 37 (14 females) AUD treatment‐seeking patients were randomized to sham or active Deep TMS. Twenty (six females) age‐matched healthy controls were included. White matter integrity was evaluated by fractional anisotropy (FA). Secondary measures included brain functional connectivity and self‐reports of craving and drinking units in the 3 months of follow‐up period.

**Results:**

White matter integrity was compromised in patients with AUD relative to healthy controls, as reflected by the widespread reduction in FA. This alteration progressed during early abstinence (3 weeks) in the absence of Deep TMS. However, stimulation of midline frontocortical areas arrested the progression of FA changes in association with decreased craving and relapse scores. Reconstruction of axonal tracts from white‐matter regions showing preserved FA values identified cortical regions in the posterior cingulate and dorsomedial prefrontal cortices where functional connectivity was persistently modulated. These effects were absent in the sham‐stimulated group.

**Conclusions:**

By integrating brain structure and function to characterize the alcohol‐dependent brain, this study provides mechanistic insights into the TMS effect, pointing to myelin plasticity as a possible mediator.

Among the population aged 15–49 years, alcohol consumption is the leading risk factor for both deaths and disability‐adjusted life‐years, with 3.8% of female deaths and 12.2% of male deaths attributable to alcohol use, according to recent data.^1^ Alcohol use disorder (AUD), the chronic excessive consumption of alcohol, is a chronic illness characterized by cycles of relapse and remission, with several associated co‐morbidities and complex neurobiological substrates of alterations. Both psychological and pharmacological interventions have shown efficacy for the treatment of AUD, but a major need for novel treatments with high patient and clinician acceptance remains.^2^

In a recent double‐blind, randomized, sham‐controlled clinical trial, we found initial support for the clinical efficacy of deep repetitive transcranial magnetic stimulation (Deep TMS) targeting the anterior cingulate (ACC) and medial prefrontal cortices (mPFC).^3^ Patients with AUD receiving Deep TMS showed significantly reduced craving and relapse compared to the sham group. Although these results support the continued development of Deep TMS as a non‐invasive treatment for AUD, the biological substrates underlying its putative efficacy are not fully understood. A mechanistic understanding of the observed Deep TMS effects may allow treatment to be optimized and predictive biomarkers to be established.

Non‐invasive brain stimulation treatments are based on the notion that abnormal neuronal activity can be retuned by entraining neuronal population activity with specific stimulation patterns.^4^, ^5^, ^6^, ^7^ Possible underlying mechanisms include the induction of activity‐dependent synaptic plasticity and changes in the intrinsic neuronal excitability.^8^ However, another form of plasticity, less frequently recognized but also activity‐dependent and therefore recruited by brain stimulation protocols, is myelin plasticity.^9^, ^10^, ^11^ Myelin plasticity refers to the dynamic regulation of myelin production by oligodendrocytes in response to increased neuronal firing.^9^ It is associated with increased axonal conduction velocity and transmission reliability.^9^ Interestingly, in *post‐mortem* tissue from patients with AUD, demyelination evidence from morphological and molecular alterations have been reported,^12^ which likely underlie the white matter alterations repeatedly found in MRI studies as a hallmark of the disease.^13^, ^14^, ^15^ White matter alterations progress during early abstinence, at least up to 6 weeks after discontinuing alcohol, suggesting a possible contribution to relapse vulnerability.^15^ We reasoned that the reduction in craving and relapse induced by Deep TMS targeting the ACC and mPFC^3^ could be in part mediated by myelin plasticity and, if so, should be reflected in the white matter microstructure as measured by diffusion‐weighted MRI.

We set out to test the above hypothesis using the same imaging framework as before^15^ to elucidate the neurobiological substrate underlying the clinical efficacy of Deep TMS targeting the frontal lobe.^3^ We first replicated the progression of white matter alterations during early abstinence in the cohort of patients with AUD, and then demonstrated that this progression was arrested by Deep TMS, specifically in active‐stimulated but not in sham‐stimulated patients, and selectively in the stimulated brain region. Persistent changes in functional connectivity were also found in the brain regions connected by the Deep TMS‐protected white matter fiber tracts. By integrating brain structure and function to characterize the alcohol dependent brain, this study provides a mechanistic insight into the effects of TMS, suggesting that its therapeutic efficacy is related to the recovery of white matter microstructure, and pointing to myelin plasticity as a possible mediator.

## Materials and Methods

### Participants

This was a double blind, sham‐controlled, randomized clinical trial performed at the Ben‐Gurion University and the Soroka Medical Centre, Be'er Sheva, Israel. Recruitment occurred between July 2016 and December 2019. The study was conducted in accordance with the Declaration of Helsinki, with approval of the local Institutional Review Board (0404–15) and the Israeli Ministry of Health and was registered at ClinicalTrials.gov (NCT02691390). The main clinical outcomes of the original study have been reported previously, where the full recruitment protocol is also described.^3^ Functional (fMRI) and structural (DTI) data were not available for all subjects in the trial. From the original study and for this secondary analysis, we derived a subset of 38 patients (19 sham‐stimulated/19 active‐stimulated) from the initial patients’ sample, all of whom possessed DTI data. One patient in the active TMS group did not complete treatment, resulting in 19 patients in the sham TMS group and 18 in the active TMS group. Age‐matched controls (*n* = 20) were also included in the trial. Participants provided written informed consent and were instructed to abstain from alcohol for 5 days before commencing treatment. The clinical assessment of the participants included in the DTI analysis is reported in Table 1 (sham *vs*. active Deep TMS) and Table 2 (AUD *vs*. Control). More details on the exclusion criteria are provided in the supporting information.

**Table 1:** Demographic characteristics of AUD patients and their group‐matched healthy controls

Characteristics	Patients [mean (SD)]	Controls [mean (SD)]	*P‐*value*
Baseline demographic characteristics			
Sample size (*n* females)	20 (7)	20 (6)	
Age	36.7 (5.8)	35.1 (7.1)	0.45
Education (years)	12.4 (1.5)	13.6 (5.4)	0.01^†^
Baseline clinical characteristics			
MoCA	28.3 (1.5)	29 (2.1)	0.26
AUDIT	26.5 (5.4)	2.8 (1.9)	<0.001^†^
ADS	19.05 (7.3)	2.2 (4.3)	<0.001^†^
TLFB (%pHDD)	32.2% (23.5)	2.4% (6.6)	<0.001^†^
PACS	17.05 (6.7)	1.4 (1.9)	<0.001^†^
BDI	14.3 (9.5)	3.3 (4.6)	<0.001^†^
CPRS‐SA			
Depression	5.4 (3.4)	2.1 (2)	0.002
Anxiety	6.9 (5)	3 (2.5)	0.012^†^
NEO‐FFI			
Neuroticism	1.9 (0.9)	1.5 (0.8)	0.15
Extraversion	2.3 (0.6)	2.4 (0.4)	0.68^†^
Openness	2.1 (0.4)	2.3 (0.3)	0.11
Agreeableness	2.5 (0.6)	2.6 (0.5)	0.83
Conscientiousness	2.6 (0.7)	3.01 (0.4)	0.07^†^

Abbreviations: ADS, Alcohol Dependence Scale; AUDIT, alcohol use disorder identification test (scores of >20 indicate high‐likelihood of dependence); CPRS, Comprehensive Psychopathological Rating Scale; MoCA, Montreal Cognitive Assessment; NEO‐FFI, abbreviated five factor personality assessment; SD, standard deviation; TLFB, timeline follow back.

* Two‐tailed *P*‐value using independent‐samples *t*‐test.

^†^ Mann–Whitney Test.

**Table 2:** Demographic characteristics of active and sham Deep TMS groups

Characteristics	Active [mean (SD)]	Sham [mean (SD)]	*P‐*value*
Baseline demographic characteristics			
Sample size (*n* females)	18 (7)	19 (7)	
Age	42.3 (9.1)	43.8 (9.5)	0.61
Education (years)	12.3 (1.6)	12.2 (3.6)	0.93
Baseline clinical characteristics			
MoCA	28.2 (1.5)	27.5 (1.5)	0.18
AUDIT	25.7 (6.8)	25.6 (6.7)	0.96
ADS	17.9 (7.3)	17.5 (6.7)	0.87
TLFB (%pHDD)	44.6% (33)	32.1% (23)	0.19
Consumption (average g/day)	69.3 (53.2)	55.03 (39.8)	0.35
PACS	15.7 (5.9)	16.5 (7.3)	0.71
BDI	14.4 (9.8)	15.2 (8.1)	0.78
SETS	23 (8.2)	24.6 (8.1)	0.54
CPRS			
Depression	5.9 (3.8)	6.3 (4.05)	0.76
Anxiety	6.9 (5.4)	8.07 (4.6)	0.5
NEO‐FFI			
Neuroticism	2.01 (0.8)	1.9 (0.8)	0.74
Extraversion	2.3 (0.6)	2.06 (0.4)	0.25
Openness	2.09 (0.4)	2.1 (0.5)	0.86
Agreeableness	2.6 (0.6)	2.5 (0.5)	0.69
Conscientiousness	2.5 (0.6)	2.6 (0.5)	0.6
Other clinical characteristics			
RMT (% of stimulator power output)	66.1% (7.5)	66.6% (8.1)	0.82
Blinding assessment (% of patients who correctly guessed their treatment arm at the end of follow‐up)	(9/17) 527%	(3/15) 20%^†^	0.82^‡^
Adverse events Headache (moderate to severe)	*n* = 2	*n* = 3	0.67^§^

Abbreviations: ADS, Alcohol Dependence Scale; AUDIT, alcohol use disorder identification test (scores of >20 indicate high‐likelihood of dependence); CPRS, Comprehensive Psychopathological Rating Scale; MoCA, Montreal Cognitive Assessment; NEO‐FFI, abbreviated five factor personality assessment; RMT, resting motor threshold; SD, standard deviation; SETS, Stanford Expectations of Treatment Scale; TLFB, timeline follow back.

* Two‐tailed *P*‐value using independent‐samples *t*‐test.

^†^ Data missing for two participants.

^‡^ χ‐Test.

^§^ Z‐Test.

### Magnetic resonance imaging

Imaging protocols were coordinated with the SyBil‐AA (Systems Biology of Alcohol Addiction) Horizon 2020 Consortium. Imaging was performed using a Philips Ingenia 3 Tesla MRI scanner (Philips Healthcare, Best, The Netherlands) equipped with a 32‐channel Philips dS Head head‐coil. Blood oxygen‐level‐dependent (BOLD) data were acquired with an echo‐planar imaging (EPI) sequence: TR = 2000 ms; TE = 30 ms; flip angle = 77°; field‐of‐view = 220 × 220; in‐plane resolution = 3.4 × 3.4 mm; slice thickness = 4 mm, no slice gap; number of axial slices (angled with the AC‐PC line) = 32; number of volumes = 360. The two collected resting state runs (baseline and follow‐up) each lasted for 12 min. A high‐resolution 3D T1‐weighted Turbo Field Echo scan was acquired before the EPI data acquisitions TR = 7.0 ms; TE = 3.2 ms; flip angle = 8°; field‐of‐view = 256 mm × 256 mm × 170 mm; voxel resolution = 1 mm × 1 mm × 1 mm; no slice gap; plane: sagittal; number of sagittal slices = 170.

Multi‐shell diffusion MRI acquisition protocol consisted of images with 30 uniformly distributed diffusion directions, acquired with b‐value of 1000 s/mm^2^, and 60 uniformly distributed diffusion directions, acquired with b‐value of 2000 s/mm^2^, in addition to a non‐weighted image (b0). Echo Planar Imaging spin‐echo diffusion sequence was used with the following parameters: TR = 7 s, TE = 108 ms, matrix size = 224 × 224 × 125, and a spatial resolution of 1.75 mm × 1.75 mm × 2.5 mm. We initially collected fMRI data, followed by the acquisition of diffusion MRI data.

Preprocessing and statistical analysis of resting state data were performed with the Analysis of Functional Neuro Images (AFNI) software v18.3.16.^16^ First, a Freesurfer‐based parcellation was performed on T1‐weighted data using the function *recon‐all*, and later used for tissue‐based regression to allow for modeling of signal fluctuations not due to BOLD signal. BOLD signal was de‐spiked, slice‐time and motion‐corrected, and then spatially transformed to the Montreal Neurological Institute (MNI) template space, using a combination of linear and non‐linear transformations. Motion censoring was set at 3 mm, and outlier fraction was set at 0.05, so that volumes exceeding these values were not included in the time‐series regression. A regression on BOLD time‐series data was performed using the *3dDeconvolve* function, and head motion effects were accounted for by adding the motion parameters and their derivatives as regressors of no interest. Afterward, the covarying signals between gray and white matter regions were removed from the residuals using the data‐driven APPLECOR method.^17^

### Microstructural data processing and statistics

All raw data were first denoised using a method based on statistical independence^18^ and then converted to ExploreDTI^19^ format. Each dataset underwent subject motion and Eddy‐current‐induced distortions corrections, followed by brain extraction to eliminate non‐brain tissue. Images were split according to their b‐value. Diffusion‐weighted data with b‐value = 1000 (30 directions in total) were used for diffusion tensor‐DTI estimation, using the robust model fitting.^20^ After the tensor model was fitted, Fractional Anisotropy (FA) maps were generated for the voxel‐wise statistical analyses.

For both cross‐sectional and longitudinal analyses, a Tract‐based spatial statistics^21^ approach was used. For cross‐sectional analysis, FA maps from AUD cohort at the first time point (TP1) and healthy controls were registered to MNI standard template ***via*** a combination of linear and nonlinear registration implemented in the Advanced Normalization Tools‐ANTs^22^; then, the FA maps in standard space were used to generate the white matter skeleton, where the FA images were projected. For longitudinal analysis, for each subject in the AUD cohort, FA maps at the second time point (TP2) were registered to FA maps at TP1 by linear registration, then brought to standard space by applying the same transformation and field warping obtained from nonlinear registration at time point TP1. Then, the FA maps at TP1 in standard space were used to generate the white matter skeleton, where the difference between FA images at the two‐time points was projected. Skeletonized FA images were tested using the randomise tool for statistical permutations testing, as part of the FSL package.^23^ Using a general linear model (GLM) design, threshold‐free cluster enhancement option was used in the statistics.

Three kinds of statistical comparisons were run. To localize differences in FA at TP1 between AUD and control cohorts, a GLM model compared FA maps between groups, while accounting for age and gender. To test the longitudinal evolution of FA between TP1 and TP2, a GLM model tested for positive or negative differences in FA maps between time points. Finally, to highlight differences in FA progression in sham *vs*. active Deep TMS, a GLM model tested for group differences in the FA progression between time points (TP2 – TP1), while accounting for age and gender.

### Tractography and DTI‐driven functional connectivity analysis

The constrained spherical deconvolution^24^ algorithm incorporated in ExploreDTI was applied to diffusion‐weighted data with b‐value = 2000 (60 directions in total) to generate whole‐brain tractography for each subject belonging to the AUD group. Based on the statistical outcome of the previous voxel‐wise analysis, highlighting a significant cluster of voxels contained within the Deep TMS stimulated area in which FA progresses differently in the sham and active groups, we defined a generative seed and selected the white matter tracts passing through this Deep TMS‐sensitive spot. All the tracts in individual spaces were registered to standard space and then averaged. A threshold was applied to get tracts that are present in at least 70% of all the subjects. Averaged tracts were masked to get the intersection between generated tracts and the gray matter standard mask. As such, we defined four cortical targets that are connected by tracts passing through the Deep TMS‐sensitive spot. These targets were used to define the seed regions for the functional connectivity analysis at post‐treatment. Seeds consisted of 5 mm radius spheres centered on the MNI coordinates of the DTI‐derived targets. From the original study,^3^ we utilized fMRI data from subjects that survived motion censoring and completed pre‐ and post‐treatment sessions (sham *n* = 18 and active *n* = 19). In this sample, two subjects from the sham group and two from the active group did not contribute DTI data. However, we retained these individuals in the analysis to maximize statistical power. To assess seed‐to‐whole brain gray matter connectivity, we conducted a regression analysis, incorporating the seed time course as a predictor variable. The analysis was performed using the *3dDeconvolve* software. The resulting 3D volumes with beta coefficients for each seed location were then compared between groups at post‐treatment, using the AFNI function 3dttest++. Results were thresholded at a per‐voxel *P* = 0.002, and multiple comparisons corrected at *alpha* = 0.05.^25^ The cluster size threshold for multiple comparison correction was estimated by entering spatial smoothness parameters of the residuals in the 3dClustsim simulation function, and as simulation mask, a gray matter mask, which consisted of the union of 80% of the subjects' EPI‐masks surviving censoring. In order to assess the potential effects of time, beta coefficients from significant clusters were extracted and entered in a 2 × 2 repeated measures ANOVA with factors time (pre/post) and group (sham/active Deep TMS).

## Results

### Progression of FA decrease in early abstinence

**Fig. 1:**
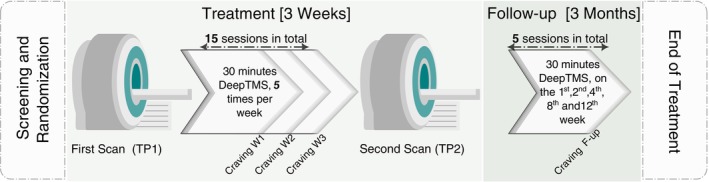
Study design. Patients with AUD are randomly assigned to sham‐ or active‐stimulated groups. All patients receive two MRI sessions (TP1 and TP2) separated by the 3 weeks of Deep TMS (active or sham) treatment (five daily sessions per week). MRI sessions included functional, anatomical and diffusion‐weighted imaging sequences. Additional Deep TMS sessions were applied during the follow up visits taking place 1, 2, 4, 8 and 12 weeks after the end of the acute daily treatment period. Craving and relapse were monitored every week during the acute treatment period and every visit during the follow up period.

**Fig. 2:**
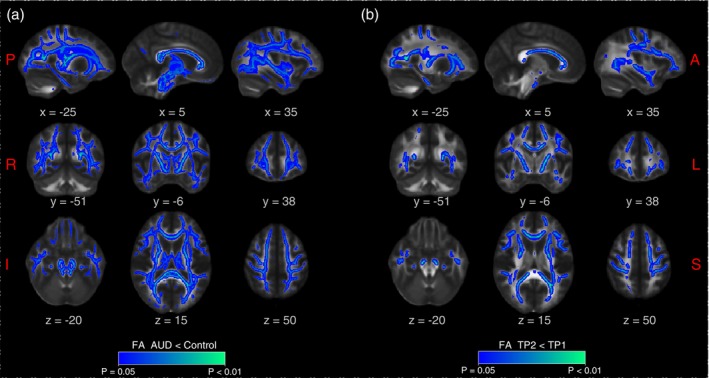
White matter alterations progress during early abstinence. Tract‐based spatial statistics showing in blue the white matter skeleton where FA is reduced in the following conditions: (a) AUD at TP1 (*n* = 20) compared to group‐matched healthy controls (*n* = 20; see Table 1 for demographic characteristics), (b) AUD at TP2 compared to TP1 (*n* = 19; longitudinal comparison) from the sham‐stimulated group of patients.

When comparing FA maps acquired in the control group and the age‐ and gender‐matched AUD cohort before treatment, we detected significantly lower FA in patients with AUD (*P* < 0.05, mean effect size d = 1.0) in most portions of the white matter skeleton, as shown in Fig. 2a. In the AUD cohort receiving sham treatment only, when comparing the progression of FA changes between time points TP1 and TP2 (Fig. 1), we detected a significant FA reduction in most areas of the white matter skeleton (*P* < 0.05, mean effect size d = 0.317), as shown in (Fig. 2b). These results replicate previous findings on white matter alterations in AUD,^14^, ^15^, ^26^ and its progression during early abstinence.^15^

### Deep TMS halts the progression of DTI alterations in early abstinence

**Fig. 3:**
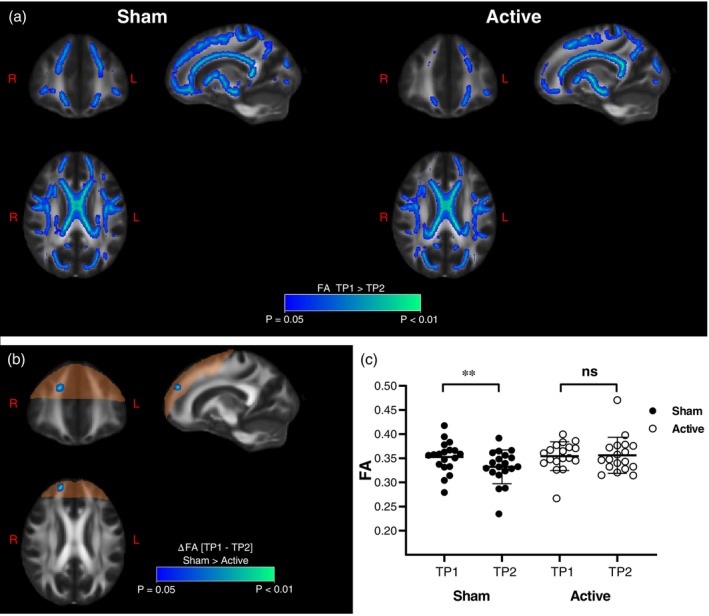
Progression of white matter alterations are arrested by Deep TMS. (a) Progression of FA between time points calculated separately for sham (left) and active (right) groups. The portion of the brain within the theoretical Deep TMS field intensity above the average threshold for action potential firing is highlighted in orange. (b) Tract‐based spatial statistics showing regions of the white matter skeleton with significant differences between active and sham Deep TMS groups in the FA change between TP2 and TP1. (c) Mean FA calculated for the two time points and for the two groups in the area of significant time × group interaction according to tract‐based spatial statistics (shown in b.). Two‐way ANOVA yielded significant time × group interaction F_1,35_ = 5.3, *P* = 0.027, then Sidak's multiple comparisons test showed significant change in Sham group only (*P* = 0.008). ns: not statistically significant, **P < 0.01.

When comparing FA changes longitudinally in the two groups (TP2 *vs*. TP1 in active and sham Deep TMS groups independently), we found a strong reduction in the extension of white matter affected by the progression of FA changes, specifically in the active Deep TMS group, and involving the right frontal lobe (Fig. 3a). We then compared the change in FA (TP2‐TP1) between the sham and active Deep TMS groups and found a significant difference in a white matter region belonging to the right frontal lobe (FroL), as shown in (Fig. 3b). In both cases, the regions with preserved FA values in the active group were entirely contained by the volume of brain tissue targeted by the Deep TMS with voltage intensities above 100 V/m, known to be suprathreshold for action potential firing (orange shading in Fig. 3). We then used the mask of the tract‐based spatial statistics (TBSS)‐defined spot (Fig. 3b) to calculate the mean FA values for each subject and compare the distributions across groups. As reported in (Fig. 3c) and (Table S1), FA reduction during abstinence is significant in the sham group (*P* < 0.002), as expected for the progression of microstructural changes, while in the active Deep TMS group, FA is stable after 3 weeks of treatment (*P* = 0.82), indicating that Deep TMS successfully prevented progression. This result remained significant when controlling for baseline differences in FA values.

To obtain insights into the lateralized effect of Deep TMS, we took the area of maximal statistical significance in the right FroL (Fig. 3b) and its counterpart in the left hemisphere (Fig. S1) and calculated the effect size (Cohen's d) of the FA reduction in AUD *vs*. healthy controls. We found significantly larger effect sizes in the right FroL compared to the left (Fig. S1), pointing to a higher vulnerability of white matter microstructure in this hemisphere, at least in the frontal region.

### Microstructural changes are paralleled by significant clinical outcome

**Fig. 4:**
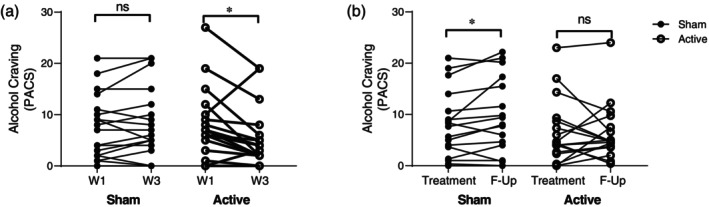
Deep TMS decreases alcohol craving. (a) Evolution of PACS (Penn Alcohol Craving Scale) values across groups (sham and active) and treatment time points (week 1 and 3). (b) Average PACS values measured during the 3 weeks treatment (“Treatment”) and at 3 months follow up (“F‐Up”) for sham and active Deep TMS groups. Wilcoxon rank test. ns: not statistically significant, *P < 0.05.

As indicated above, the sample of subjects used in this study is slightly different from the originally reported cohort,^3^ due to requirements in the quality of the diffusion‐weighted images. Therefore, we reanalyzed the clinical outcome of Deep TMS with the present active and sham Deep TMS groups. As previously reported,^3^ 3 weeks of Deep TMS treatment reduced craving levels, specifically in the active group (Fig. 4a). Furthermore, craving levels remained lower in the active group 3 months after Deep TMS treatment but not in the sham group, in which craving increased over the follow‐up period (Fig. 4b). In good agreement, the average‐drinking units (DU) in the follow up period was lower for active *vs*. sham Deep TMS‐treated patients (Fig. S2).

### Functional connectivity is modulated in the cortical regions defined by the Deep TMS‐sensitive axonal tracts

**Fig. 5:**
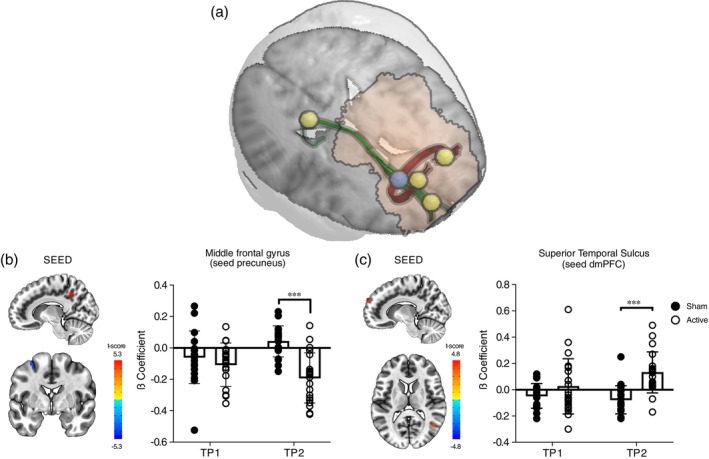
White matter tracts protected by TMS define terminal fields in the gray matter that show persistent changes in functional connectivity. (a) Cortical targets (yellow spheres) defined by the terminations of white matter streamlines (green: ipsilateral/association; red: bilateral/commissural) passing through the Deep TMS‐sensitive spot (blue sphere). Two fiber tracts were consistently identified. (b) Increased negative functional connectivity in the active group at TP2 between the PCCs (defined by the green tract) and middle frontal gyrus. (c) Increased positive functional connectivity in active Deep TMS *vs*. sham at TP2 between the dmPFC (defined by the green tract) and the pSTS.

Tractography was used to trace streamlines passing through the white matter spot sensitive to Deep TMS and shown in Fig. 3b. Two fiber tracts were consistently identified across subjects, one of association and one of commissural fibers (green and red tracts, respectively, in Fig. 5a) which were present in at least 70% of the subjects. The terminal fields of these tracts defined four regions of interest in the gray matter. The posterior cingulate cortex (PCC; MNI: 12, −50, 40) and the dorsomedial PFC (dmPFC; MNI: 11, 62, 28), defined by the association fibers and located in posterior and anterior portions of the default mode network, respectively (Fig. 5a); and bilateral regions of the dorsolateral PFC (dlPFC; MNI: 10, 56, 42 and −13, 55, 34), connected by the commissural fibers (Fig. 5a).

Functional connectivity analysis using these regions as seeds showed significant main effects of the group at post‐treatment. Follow‐up ANOVAs on extracted beta coefficients showed time × group interactions driven by between‐group differences at post‐treatment. Specifically, we found increased negative connectivity in active *vs*. sham Deep TMS groups between PCC and the middle frontal gyrus, in the premotor cortex (MNI = −32, −2, 58; 6 voxels; main effect of group F_1,35_ = 16.5, *P* < 0.001, np^2^ = 0.32; time × group interaction F_1,35_ = 10.9, *P* = 0.002, np^2^ = 0.24; between‐group difference at post‐treatment, *P* < 0.001, pre‐treatment *P* = 0.33). The results were not affected by removal of one outlier (between‐group difference at post‐treatment, *P* < 0.001, pre‐treatment *P* = 0.09; Fig. 5b). Also, we found increased positive connectivity in active *vs*. sham Deep TMS groups between the dmPFC seed and the posterior portion of the superior temporal sulcus (pSTS, MNI = 43, −59, 13; 6 voxels; main effect of group F_1,35_ = 12.5, *P* = 0.001, np^2^ = 0.26; time × group interaction F_1,35_ = 5.3, *P* = 0.028, np^2^ = 0.13; between‐group difference at post‐treatment, *P* < 0.001, pre‐treatment *P* = 0.17). The results were not affected by the removal of one outlier (between‐group difference at post‐treatment, *P* < 0.001, pre‐treatment *P* = 0.33; Fig. 5c).

## Discussion

The fundamental finding of this study is that a protocol of Deep TMS, targeting midline structures of the frontal lobe in patients with AUD to achieve a positive clinical outcome, also had a positive impact on white matter microstructure, which in turn related to persistent changes in functional connectivity. More specifically, we show that the progressive reduction in FA in the white matter of patients with AUD that occurs during early abstinence (see Ref. 15 and confirmed here, Fig. 2) can be arrested by high frequency (10 Hz) Deep TMS applied during 3 weeks of treatment. Furthermore, we show that this structural effect is accompanied by persistent functional changes in the networks connected by the tracts protected by Deep TMS, including the PCC, dmPFC, and dlPFC. Importantly, the same group of actively stimulated patients showed a reduction in craving and relapse scores.^3^ Our results draw attention to the possibility that induction of white matter plasticity, potentially driven by axonal firing, contributes to the therapeutic effect of Deep TMS in AUD.

### Progression of microstructural alterations in white matter

Preventing relapse to heavy drinking is the primary goal of AUD treatment and is particularly challenging in the first months following cessation of use.^27^ We recently carried out a translational study in patients with AUD and in a rat AUD model, and found a progression of microstructural alterations in white matter that continued for at least 6 weeks after discontinuation of alcohol use.^15^ This unexpected result identified a process that appears to be triggered by, or persist after cessation of alcohol use, and that could contribute to the high relapse rates in early abstinence.^27^ In our previous study,^15^ mathematical modeling of water diffusion in distinct biological compartments suggested that this FA reduction could be explained by a decrease in myelin content and/or a glial reactivity in the white matter,^15^ two plausible neurobiological mechanisms in AUD.^28^ We now replicate this result in an independent patient cohort and using a different MRI acquisition protocol. The robustness of this finding is particularly valuable against the background of emerging insights into limited reproducibility in many types of MRI studies with conventional sample sizes.^29^

The progression of FA changes observed in sham‐treated patients was arrested by 3 weeks of active Deep TMS targeting midline structures of the frontal lobe. The Deep TMS‐protected white matter regions belonged to frontal lobe fibers, including both association and commissural pathways, and were located in the area of the brain where Deep TMS was expected to produce suprathreshold activation for action potential firing (orange shadow in Fig. 3). Overall, these results provided strong support to the specificity of the finding.

Lateralization of FA changes to the right hemisphere in abstinent AUD subjects has previously been reported in the frontal lobe.^30^ Here, we replicated this observation, and showed right lateralization of microstructural white matter changes both for the decrease in FA in patients with AUD *vs*. healthy controls (Fig. 2a), and for the protection exerted by Deep TMS within the former group (Fig. 3). The lateralization of these two phenomena may be related. For instance, the lower FA values in the right hemisphere of patients with AUD *vs*. healthy controls (Fig. S1) may have increased the statistical sensitivity to detect a positive TMS effect. Lateralization has also been reported in neuroimaging studies of cue reactivity, including in a meta‐analysis,^31^ but fully consistent evidence is not yet available.

### What neurobiological process mediates the measured microstructural and clinical improvement?

TMS has demonstrated clinical efficacy on several indications,^32^ but a mechanistic understanding of these effects is lacking at the level of molecular and cellular physiology. A mechanism of action that has been hypothesized in the context of psychiatric disorders in general, and addiction in particular, is modulation of neuronal activity in brain circuits proposed to be relevant for different psychological processes (i.e., salience of, and reactivity to drug‐associated cues, response inhibition), and modulation of neurotransmitters activity (especially dopamine and glutamate).^5^ However, in addition to neuron‐centric interpretations, recent evidence suggests the possibility of other potentially contributing mechanisms.^33^ By increasing neuronal firing rates,^5^ high‐frequency TMS may recruit activity‐dependent increases in myelination, thereby increasing axonal conduction velocity and transmission reliability between brain areas.^33^ Previous work has shown increases in FA after TMS treatment in patients with major depression.^34^, ^35^ Our results show a rescue of FA values in white matter tracts connecting structures with a key role in alcohol addiction, such as the dlPFC, dmPFC and PCC,^36^, ^37^, ^38^ together with signals of decreased craving and relapse. Together, these observations lend support to the possibility that activity‐dependent myelination and concomitant rescue of structural connectivity contribute to the therapeutic efficacy of Deep TMS in AUD.

Adaptive myelination, or myelin plasticity, is the process by which electrical activity in axon fibers instructs oligodendrocytes to myelinate active axons, and oligodendrocyte precursor cells (OPCs) to produce more mature oligodendrocytes, increasing axonal conduction velocity.^39^ Even at low intensity, repetitive TMS has been shown to increase the number of newborn oligodendrocytes in the adult mouse cortex, without altering oligodendrogenesis but rather increasing cell survival and enhancing myelination.^40^ In this study, the authors further reported increases in myelin internode length induced by TMS,^40^ which would translate into faster action potential conduction velocity.^41^ In this manner, TMS could serve to repair, at least partially, myelin deficits induced by withdrawal from prolonged alcohol consumption.^12^ Of note, the vesicular release of glutamate from the axon within white matter is known to initiate the signaling cascade coupling neuronal activity to OPCs response,^42^, ^43^ while chronic drug use is known to dysregulate glutamate homeostasis in the brain.^44^, ^45^ This opens the possibility that dysfunctional myelin plasticity is a common pathophysiological element of addiction.

In addition, TMS could influence white matter microstructure in patients with AUD by targeting other glial cell types in the stimulated area. TMS has been shown to induce a transient increase in GFAP expression *in vivo*,^46^ and inhibit neurotoxic polarization of astrocytes.^34^ Application of very low‐intensity, but high‐frequency TMS following an ischemic injury, or induction of demyelination, appears to induce a reactive microglia phenotype.^46^, ^47^ In contrast, high‐intensity, high‐frequency TMS attenuates microglial reactivity.^48^ Importantly, inflammation has long been postulated as a contributor to alcohol pathophysiology,^49^ with inflammatory markers correlating with the lifetime of alcohol consumption and age of drinking onset.^28^ We have reported sustained microglia reactivity during early alcohol abstinence in an animal model of AUD, with a change in cellular morphology consistent with neuroinflammation, and reflected in diffusion‐weighted MRI parameters.^50^ The latter finding was translated to patients with AUD.^50^ We recently showed that diffusion‐based MRI is sensitive to changes in glia morphology typical of inflammatory conditions, and not only to changes in the myelin content.^51^ It is possible that at least part of the benefit of the TMS treatment might be associated with a reduction of pro‐inflammatory activation of glial cells. Overall, it seems important to take into account not only the indirect stimulation of myelin plasticity processes *via* heightened action potential firing but also the direct effects of TMS on glial cells.

### Linking structural and functional changes to explain positive clinical outcomes

Our previous analysis of functional connectivity in this cohort used hypothesis‐driven selection of seed‐regions for resting‐state fMRI analysis. It found persistent changes in functional connectivity of the mPFC, corresponding to the anterior default mode network, and the dorsal anterior cingulate cortex (ACC), corresponding to the salience network. Connectivity between mPFC and subgenual ACC was decreased by active Deep TMS, as well as between the dorsal ACC and the caudate.^3^ In the present study, we analyzed the connectivity of gray matter areas identified as projections of axonal tracts selected based on Deep TMS results. This data‐driven approach found four cortical targets. Two of them, the PCC and dmPFC connected through association fibers in the right hemisphere, and the other two corresponded to bilateral regions of the dlPFC connected through commissural fibers. All the regions found in the previous^3^ as well as the present analysis, have previously been implicated in AUD and are involved in cognitive functions such as inhibitory control, decision‐making, planning, cognitive flexibility, and working memory.^52^, ^53^ Recent work studying brain lesions associated with remission of nicotine or alcohol addiction elegantly showed a set of brain regions and tracts conforming an addiction remission network.^54^ Interestingly, the brain regions and tracts protected by Deep TMS stimulation in our study were well contained by this network, linking the structural and functional changes found (Figs 3 and 5) to the observed clinical outcome (Fig. 4 and Ref. 3), and suggesting that the mechanism identified here could generalize to other addictions.

Our fMRI results should be interpreted with caution due to the limited sample size. Nevertheless, they provide support for a functional impact of the identified microstructural changes in white matter. As previously reported,^3^ functional connectivity changes found in the active Deep TMS group opposed those commonly reported in heavy alcohol users and in AUD.^55^, ^56^, ^57^, ^58^ A significant increase in functional connectivity, with negative beta coefficients, was found in active Deep TMS participants between the PCC and a cluster in the middle frontal gyrus and, more specifically, in the frontal eye field (FEF) region of the premotor cortex. The PCC is the posterior node of the default mode network (DMN), a network involved in self‐referential processing, task disengagement and “mind‐wandering”.^59^ On the contrary, the FEF is one of the nodes of the dorsal attention network (DAN), a network involved in voluntary response selection during attention‐demanding tasks.^60^ Typically, the DMN and DAN networks are anti‐correlated as they reflect competing cognitive processes, and their spontaneous interaction is crucial in guiding stimulus‐independent *versus* attention‐driven thought.^61^, ^62^ The increased anticorrelation between DMN and DAN regions in the active group, indicates potential improvement in spontaneous shifting between competing internally *versus* externally oriented states.

We also found an increase in functional connectivity between the dmPFC and pSTS. This is a central connection in the so‐called social network, and together with the PCC has been postulated to be involved in internalization, strategic planning, and decision‐making in social contexts.^63^ The dmPFC seems to store longer‐term representations of social interactions and guides behavioral strategy, while pSTS integrates visuospatial information, encodes contextual updates and temporary goals.^64^, ^65^, ^66^ Increased connectivity between these regions may reflect an improvement in social flexibility and the adaptation to contingency changes in social interactions. Whether the strengthened coupling in this network improves social abilities in the patients and contributes to the therapeutic action of Deep TMS, is an appealing interpretation that will deserve further investigation.

### Limitations

Our study has several limitations discussed in the previous work.^3^ First, to definitively establish clinical efficacy of Deep TMS will require replication in a multicenter study with larger sample sizes. Second, although both male and female patients entered the study, the size and sex composition of the sample did not allow for an analysis of potential sex effects, neither on white matter alterations nor the clinical effects. Finally, it is possible that the stimulation protocol could benefit from optimization, depending on the type of process that is most important for the clinical effects. Different neurobiological mechanisms underpinning the observed clinical efficacy of Deep TMS, such as activation of specific neuronal networks, induction of myelin plasticity, or potential anti‐inflammatory effects, may benefit from different optimization parameters. Translational work in animal models may help dissect the different contributions to Deep TMS effects and may help elucidate optimal stimulation protocols.

## Conclusions

Despite the limitations discussed above, we believe that the findings presented here represent an important advance. We have combined structural and functional neuroimaging to identify a potential basis for the positive effect of Deep TMS in AUD. We show that Deep TMS is able to arrest the progression of microstructural alterations in white matter with a sustained impact on functional connectivity. A potential broader implication of our findings is that positive effects on white matter microstructure should be considered as potential mediators of Deep TMS effects and that specific effort should be made to identify optimal conditions for achieving this type of effect.

## Disclosure statement

AZ is an inventor of deep TMS coils and has a financial interest in BrainsWay, which produces and markets these coils. MHe has received consulting fees, research support, or other compensation from Indivior, Camurus, BrainsWay, Aelis Farma, and Janssen Pharmaceuticals. All other authors report no biomedical financial interests or potential conflicts of interest.

## Author contributions

Mohamed Kotb Selim, Silvia De Santis, Irene Perini and Markus Heilig analyzed the data, Markus Heilig, Abraham Zangen, Wolfgang H. Sommer and Santiago Canals supervised the project, Mohamed Kotb Selim, Silvia De Santis and Santiago Canals wrote the first manuscript draft, and all authors reviewed it.

## Supporting information


**Fig. S1.** Effect size in the significant spot (FroL). (a) comparison of effect sizes in the AUD *vs*. healthy control contrast, measured by Cohen's‐d metric at the significant spot (FroL right) and its contralateral counterpart (FroL left), (b) Anatomical presentation of both clusters. unpaired t‐test ***P < 0.001.
**Fig. S2.** Comparison of alcohol consumption during the follow‐up period. Average drinking units (DU) in the follow up period was lower for active *vs*. sham Deep TMS‐treated patients. One DU = 12g of ethanol. Mann–Whitney test *P < 0.05.
**Table S1.** Raw values of Fractional Anisotropy encapsulated in the significant treated spot (FroL), in both groups; Active and Sham.

## Data Availability

The data that support the findings of this study are available from the corresponding author, SC, upon a reasonable request.
