# Supplementary material for: Repetitive deep TMS in alcohol dependent patients halts progression of white matter changes in early abstinence
Source: Psychiatry Clin Neurosci. 2023 Dec 12;78(3):176–85. doi: 10.1111/pcn.13624 (PMC11488632; doi:10.1111/pcn.13624)
Supplement: Supplementary file 1 — Fig. S1. Effect size in the significant spot (FroL). (a) comparison of effect sizes in the AUD vs. healthy control contrast, measured by Cohen's‐d metric at the significant spot (FroL right) and its contralateral counterpart (FroL left), (b) Anatomical presentation of both clusters. unpaired t‐test ***P < 0.001. Fig. S2. Comparison of alcohol consumption during the follow‐up period. Average drinking units (DU) in the follow up period was lower for active vs. sham Deep TMS‐treated patients. One DU = 12g of ethanol. Mann–Whitney test *P < 0.05. Table S1. Raw values of Fractional Anisotropy encapsulated in the significant treated spot (FroL), in both groups; Active and Sham. [file PCN-78-176-s001.docx]

**Supporting Information**

**Figure S1. Effect size in the significant spot (FroL).** **A**. Comparison of effect sizes in the AUD vs. healthy control contrast, measured by Cohen's-d metric at the significant spot (FroL right) and its contralateral counterpart (FroL left), **B.** Anatomical presentation of both clusters. unpaired t-test *** p<0.001.


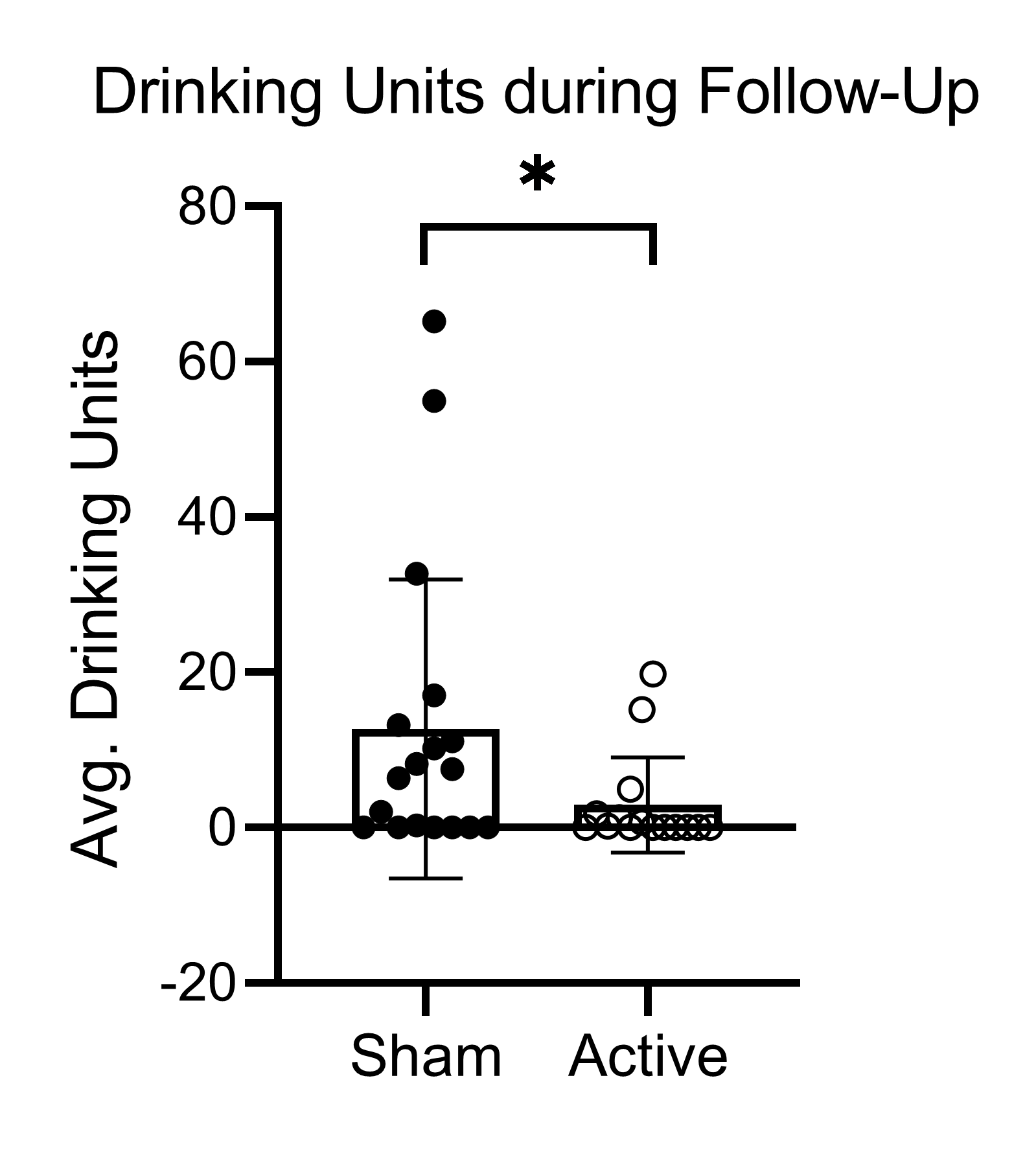


**Figure S2. Comparison of alcohol consumption during the follow-up period**. Average drinking units (DU) in the follow up period was lower for active vs. sham Deep TMS-treated patients. One DU=12g of ethanol. Mann-Whitney test *p<0.05.

| **FA (FroL) Active-TP1** | **FA (FroL) Active-TP2** | **FA (FroL) Sham-TP1** | **FA (FroL) Sham-TP2** |
| --- | --- | --- | --- |
| 0.266909 | 0.314349 | 0.347316 | 0.366215 |
| 0.367286 | 0.470511 | 0.313917 | 0.234664 |
| 0.370658 | 0.357384 | 0.394509 | 0.339525 |
| 0.372684 | 0.372015 | 0.383116 | 0.361168 |
| 0.326819 | 0.320581 | 0.331703 | 0.328488 |
| 0.347684 | 0.376477 | 0.366054 | 0.355961 |
| 0.343613 | 0.327845 | 0.417745 | 0.391775 |
| 0.377182 | 0.375534 | 0.358617 | 0.323576 |
| 0.345012 | 0.332329 | 0.337254 | 0.34102 |
| 0.385728 | 0.3979 | 0.362842 | 0.348147 |
| 0.350604 | 0.34751 | 0.350428 | 0.332119 |
| 0.360555 | 0.34619 | 0.377826 | 0.32923 |
| 0.349888 | 0.31481 | 0.279123 | 0.28664 |
| 0.383118 | 0.362787 | 0.304056 | 0.288118 |
| 0.340117 | 0.342998 | 0.332734 | 0.3325 |
| 0.325986 | 0.340075 | 0.366755 | 0.36468 |
| 0.364859 | 0.332463 | 0.358796 | 0.315342 |
| 0.399665 | 0.378268 | 0.355919 | 0.320925 |
|  |  | 0.357449 | 0.350938 |
| Mean(SD):0.354(0.03) | Mean(SD):0.356(0.037) | Mean(SD):0.352(0.032) | Mean(SD):):0.332(0.035) |
| Paired t-test (*p*) = 0.82 | | Paired t-test (*p*) = 0.0019*** | |

**Table S1**: Raw values of Fractional Anisotropy encapsulated in the significant treated spot (FroL), in both groups; Active and Sham.

**Supplementary Methods**

**Participants Inclusion Criteria:**

• Age 18 – 65.

• Current diagnosis of moderate-severe alcohol use disorder.

• Alcohol use in the past month.

• Right-handed (self-report).

• If female, negative urine pregnancy test.

• If female, must either agree to practice an effective birth control method, agree to abstinence from intercourse, be surgically sterile or postmenopausal for at least one year.

**Participants Exclusion Criteria:**

• Currently pregnant or breastfeeding.

• More than mild cognitive impairment, as determined by a score on the Montreal Cognitive Assessment (MoCA) <25.^1^

• Current DSM-5 diagnosis of schizophrenia, bipolar disorder, or other psychotic disorder.

• Use in the past 2 weeks of medication or illicit drug with known high proconvulsant action, as self-reported or detected using urine toxicology screening and with accordance to the Physician's best judgement.

• Any history of clinically significant neurological disorders, including organic brain disease, epilepsy, stroke, brain lesions, multiple sclerosis, previous neurosurgery, or personal history of head trauma that resulted in loss of consciousness for > 5 minutes and retrograde amnesia for > 30 minutes (self-reported history),

• Any history of seizures other than febrile childhood seizures (self-reported history).

• Clinically significant hearing impairment.

• Presence of ferromagnetic objects in the body that are contraindicated for MRI of the head (pacemakers or other implanted electrical devices, brain stimulators, some types of dental implants, aneurysm clips, metallic prostheses, permanent eyeliner, implanted delivery pump, or shrapnel fragments), or fear of enclosed spaces. Eligibility will be determined by the “MRI Safety Screening Questionnaire” and verified, if necessary, by a radiology consultant. Some of the patients who will be excluded from the imaging part of the study will be included in the clinical part.

• Any psychiatric, medical, or social condition, whether or not listed above, due to which, in the judgement of the investigators and after any consults if indicated, participation in the study is not in the best interest of the patient.

**Participants Recruiting**

Participants were recruited via social media and local newspaper advertisements. All participants provided written informed consent and their anonymity was preserved. This clinical trial conforms to the provisions of the Declaration of Helsinki.

**AUD Diagnoses**

Participants were diagnosed by an independent psychiatrist to match the current DSM-5 diagnosis of moderate to severe alcohol dependence.

**Comorbidities**

Anxiety and depression are common comorbidities of AUD; they were measured and reported in the participant’s demographics table. There was a reduction in these measures following alcohol detoxification regardless of group.

**Medication Regimen**

Participants were not provided with new medication prescriptions and were advised to maintain their existing medication regimens that would not interfere with the TMS treatment.

**Duration of AUD**

The study adhered to the DSM-5 criteria for AUD, indicating that all participants had been dealing with AUD for a minimum of one year.

**Abstinence Period**

Patients had maintained abstinence for a minimum of 5 days but no longer than one month.

**Craving Assessment**

Craving was assessed using the Penn Alcohol Craving Scale (PACS) questionnaire, a tool that measures various aspects of craving experienced during the previous week. In the revised version, we have specified it in the text.

**TMS Stimulation Protocol**

Active and sham dTMS was administered using a Magstim Rapid2 TMS stimulator (Magstim Co. Ltd.) equipped with an H7 coil (BrainsWay). In each session, the optimal spot on the scalp for stimulation of the leg motor cortex was localised, and resting motor threshold (RMT) was defined as previously described^2^. Then, the coil was moved 4 cm anterior to the motor spot, aligned symmetrically (over the mPFC). Each daily stimulation session following localization over the mPFC lasted about 30 minutes and included 3000 pulses using the intensity of the leg RMT measured for that individual patient (100% of leg RMT). The session included 100 trains of pulses (3 seconds per train) at 10Hz, with an inter-train interval of 15 seconds. Placebo treatment was performed using a Sham coil located within the same helmet as the Active coil. The Sham coil is capable of producing similar acoustic artifacts and scalp sensations as the real coil, but induces only negligible electric fields in the brain^2^. We have now included this information in the supplement as requested.

**Instructions for the rs-fMRI measurements**

During resting state, the experimenter instructed the participants to keep their eyes open and “let their mind wander”, without pondering on a specific thought, and the scan included a white fixation cross in the middle of the screen. Participants were inquired whether they had fallen asleep at the end of the resting state scan (the responses were always negative) and DTI scan followed resting state.

**Supplementary References**:

1. Nasreddine ZS, Phillips NA, BÃ©dirian V, et al. The Montreal Cognitive Assessment, MoCA: A Brief Screening Tool For Mild Cognitive Impairment: MOCA: A BRIEF SCREENING TOOL FOR MCI. *J Am Geriatr Soc*. 2005;53(4):695-699. doi:10.1111/j.1532-5415.2005.53221.x

2. Roth Y, Pell GS, Chistyakov AV, Sinai A, Zangen A, Zaaroor M. Motor cortex activation by H-coil and figure-8 coil at different depths. Combined motor threshold and electric field distribution study. *Clin Neurophysiol*. 2014;125(2):336-343. doi:10.1016/j.clinph.2013.07.013
